# Laser Excision of Focal Epithelial Hyperplasia (Heck's Disease): A Rare Case Report

**DOI:** 10.5005/jp-journals-10005-1569

**Published:** 2018

**Authors:** Srinivas Nallanchakrava, Naga Sreebala, Basavaraj, Farheena Sindgi

**Affiliations:** 1-4 Department of Pedodontics and Preventive Dentistry, Panineeya Mahavidyalaya Institute of Dental Sciences and Research Centre, Hyderabad, Telangana, India

**Keywords:** Focal epithelial hyperplasia, Heck's disease, Human papilloma virus

## Abstract

Heck's disease or focal epithelial hyperplasia is a rare, benign and asymptomatic lesion of oral mucosa that is characterized by the presence of soft, sessile papules, white to pinkish in colour that occur diffusely in oral cavity. Prevalence rate of this disease greatly varies from one geographic region to another. It is found to be closely associated with human papilloma virus (HPV). This paper reports an Indian case of focal epithelial hyperplasia in a 5-year-old boy that is diagnosed according to both clinical and histopathological features, where human poillomavirus (HPV) subtype 32 was found associated with disease through polymerase chain reaction analyses. A dental practitioner should be aware of this disease and must perform a careful evaluation both clinically and histopathologically for setting the diagnosis with other diseases.

**How to cite this article:**

Nallanchakrava S, Sreebala N, Basavaraj, Sindgi F. Laser Excision of Focal Epithelial Hyperplasia (Heck's Disease): A Rare Case Report. Int J Clin Pediatr Dent, 2018;11(6):526-528

## INTRODUCTION

Focal epithelial hyperplasia (FEH) is currently known as multifocal hyperplasia. Dr Heck with his team in 1965 reported the first case on multifocal epithelial hyperplasia hence called as Heck's disease.^1,2^ The occurrence rate of this disease is seen in Eskimos, Inuits, Indians resident in Central, South and North America, and is less common in Europe and Africa.^1,2^ In Eskimos population, the prevalence rate is around 7–36%.^1^ It is commonly seen in buccal mucosa of lower lip, tongue, and less common in the upper lip and palate.^2^ The rate of prevalence of FEH is less in Asian countries.^1^ Human papillomavirus (HPV) is found to be the main etiological factor behind the disease.^2–4^ The HPV virus is a DNA virus that belongs to the Papillomaviridae family.^3^ There are more than 100 subtypes of HPV that involve the lesions of hand, feet, and genital area. It has been reported in various case reports that nonkeratinized and keratinized mucosa in the oral cavity are specifically affected with HPV 13 and 32.^3,5^ Human lymphocytic antigen (HLA-DR4 DRB1* 0404) alleles, in particular, are found associated genetically with multifocal epithelial hyperplasia,^2^ which was also stated as per the study conducted by Garcia-Corona et al. in 2004.^6^ The other risk factors that are responsible for the disease are lack of oral hygiene, low socioeconomic status, environmental and nutritional deficiency, immunocompromised patients such as HIV positive in particular who are on a high antiretroviral treatment.^2,7^

## CASE REPORT

**Figs 1A and B:**
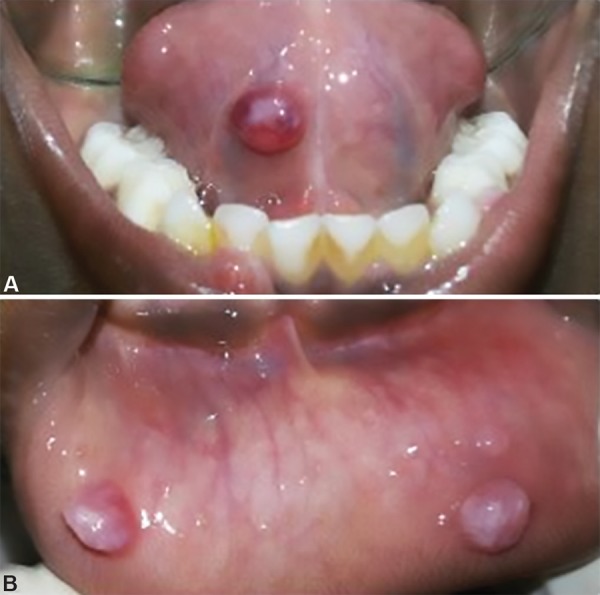
(A) Presence of papule on left ventral aspect of the tongue; (B) Lower lip showing the presence of papules on right and on left aspect

**Fig. 2:**
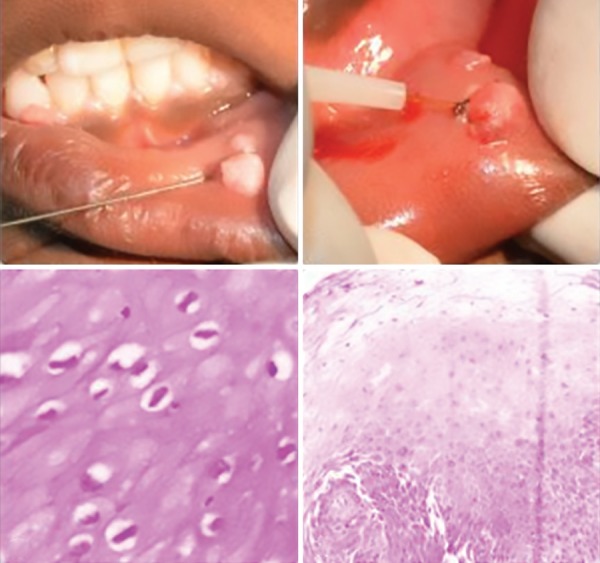
Lesions excised under local anesthesia using diode soft tissue laser

A 5-year-old boy reported to the Department of Pediatric Dentistry at Panineeya Institute of Dental Sciences, and parents give a history of multiple small swellings in child's mouth since 3 months which occurred during high fever and gradually increased in size even after fever subsides. The medical history, dental history, and family history were noncontributory. The extraoral examination did not reveal any specific finding, but intraoral examination did reveal the presence of soft, sessile papules varying 2–10 cm in dimension, two papules present on the right and left the side of the lower lip region, and one on the left ventral aspect of the tongue (Fig. 1). Based on history, clinical features and nature of lesion, a provisional diagnosis of focal epithelial hyperplasia was considered and differential diagnosis of squamous cell papilloma, condylomataacuminata, mucocele, focal dermal hypoplasia (Goltz–Gorlin syndrome) were considered. The patient was subjected to complete the hematological examination before the procedure and all the parameters were within normal limits. All the three lesions were excised under local anesthesia using diode soft tissue laser of 810 nm of 3–3.5W power for 3–60 seconds intermittently and specimens were sent for histopathology and polymerase chain reaction (PCR) evaluation (Fig. 2). The histopathology reports revealed the presence of benign parakeratotic hyperplastic mucosa with marked papillomatosis and acanthosis, and some of the cells showed isolated perinuclear vacuolization and the presence of occasional mitosoid cells. There was no evidence of dysplasia. These features were suggestive of squamous epithelial hyperplasia without atypical features of Heck's disease and further PCR was performed to confirm the subtype of virus associated with infection. Thus, this revealed the presence of HPV subtype 32 as etiology of this condition. Based on histopathology and PCR analyses, a final diagnosis as Heck's disease (focal epithelial hyperplasia) was established.

## DISCUSSION

**Figs 3A and B:**
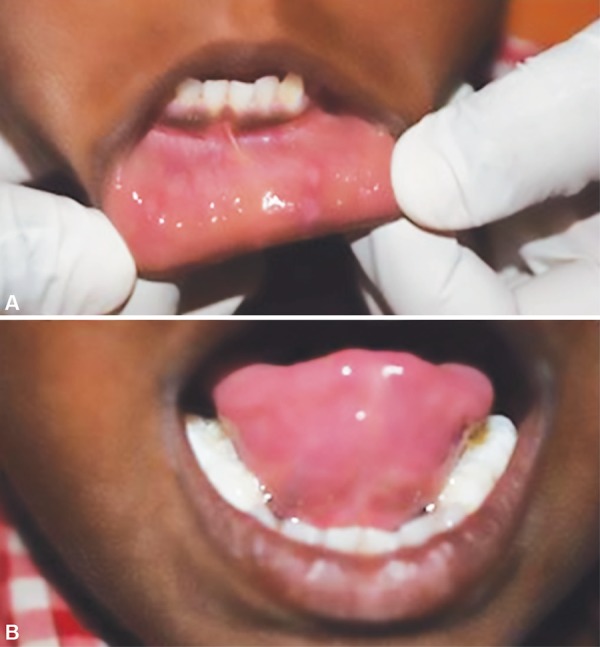
(A) Lower lip showing no regression of lesion; (B) Left ventral aspect of tongue shows good healing with no regression

Focal epithelial hyperplasia is a rare and benign condition of the mucosa which is self-limiting and requires treatment because of both functional and esthetic concerns as the lesions may interfere during a speech or esthetically unpleasing.^1,7^ Children are more commonly affected followed by middle-aged adults, and familial occurrence is also reported.^8–10^ As HPV was one of the etiological factors, the same samples were subjected to PCR analysis to identify the associated subtype of the virus. This is a rapid technique that aids in establishing the viral subtypes for Heck's disease.^6,8^ In this case, subtype 32 was observed to be associated with the disease. Histological changes are seen in the epithelial layer without affecting the connective tissue.^9^ Focal epithelial hyperplasia is reported to be a benign condition of mucosa as stated by Durso et al. but on contrary Moerman et al. stated it to be at high risk of malignant transformation.^4,10^ The characteristic feature reveals the presence of acanthosis, focal parakeratosis and verrucous proliferation with marked papillomatosis in squamous epithelium.^1^ In children, there are fair chances of regression of lesion and for esthetic and functional concern, removal of the lesion was advocated. The other proposed methods for excisions of lesions apart from laser surgery are electrodesiccation, cryosurgery or topical application of interferon beta which was suggested by Steinhoff et al.^6,8,10^ In the present case, the lesion was excised through soft tissue diode laser under controlled parameter that provided less discomfort to child as well as the clean operatory field throughout the procedure. Excision, when achieved from the laser, provides better histological results.^1,8^ Diagnosis of this disease is extremely important to rule out with other diseases such as condylomata acuminate, inflammatory fibrous hyperplasia, papillary hyperplasia, and Goltz-Gorlin syndrome.^1,8^ A thorough physical examination and investigation should be carried out for establishing a differential diagnosis.^10^ Differential diagnosis with condylomata acuminata, in particular, is essential because the clinical characteristic, appearance of lesions are same and both are associated with HPV.^9^ Hence, the PCR analysis was done to determine the subtypes of viruses and to confirm the diagnosis. In this case, the presence of HPV subtype 32 confirmed the disease as focal epithelial hyperplasia, thus, by eliminating the differential diagnosis with *condylomata acuminata* and other viral infections. Postoperative examination after one month revealed complete healing of the soft tissue from where the lesions were excised, and no new lesions were observed (Fig. 3). However, in such cases, a long term follow-up is required to rule out any further progression of the disease.

## CONCLUSION

Heck's disease, although a very rare, is benign viral infection of the oral mucosa that has its association with HPV. It is very important to get diagnosed on time, and the necessary appropriate treatment should be recommended both for functional and esthetic concerns. It is also necessary to differentiate with other viral conditions that effect the oral mucosa.

## CLINICAL SIGNIFICANCE

Human papillomavirus (HPV) which is highly contagious in nature therefore, high level care has to be taken while examing and treating such type of lesions.
